# Metabolomics and transcriptomics strategies to reveal the mechanism of diversity of maize kernel color and quality

**DOI:** 10.1186/s12864-023-09272-x

**Published:** 2023-04-12

**Authors:** Yufeng Jiang, Li Yang, Hexia Xie, Lanqiu Qin, Lingqiang Wang, Xiaodong Xie, Haiyu Zhou, Xianjie Tan, Jinguo Zhou, Weidong Cheng

**Affiliations:** 1 Maize Research Institute, Guangxi Academy of Agricultural Sciences, Nanning, 530007 China; 2 Technical Support Department of Wuhan Metware Biotechnology, Wuhan, 430075 China; 3 State Key Laboratory for Conservation & Utilization of Subtropical Agro-Bioresources, College of Agriculture, Guangxi University, Nanning, 530004 China

**Keywords:** Maize, Metabolomics, Kernel color, Nutritional quality, RNA-seq

## Abstract

**Background:**

Maize has many kernel colors, from white to dark black. However, research on the color and nutritional quality of the different varieties is limited. The color of the maize grain is an important characteristic. Colored maize is rich in nutrients, which have received attention for their role in diet-related chronic diseases and have different degrees of anti-stress protection for animal and human health.

**Methods:**

A comprehensive metabolome (LC-MS/MS) and transcriptome analysis was performed in this study to compare different colored maize varieties from the perspective of multiple recombination in order to study the nutritional value of maize with different colors and the molecular mechanism of color formation.

**Results:**

Maize kernels with diverse colors contain different types of health-promoting compounds, highlighting that different maize varieties can be used as functional foods according to human needs. Among them, red-purple and purple-black maize contain more flavonoids than white and yellow kernels. Purple-black kernels have a high content of amino acids and nucleotides, while red-purple kernels significantly accumulate sugar alcohols and lipids.

**Conclusion:**

Our study can provide insights for improving people’s diets and provide a theoretical basis for the study of food structure for chronic diseases.

**Supplementary Information:**

The online version contains supplementary material available at 10.1186/s12864-023-09272-x.

## Background

Maize, or corn (*Zea mays* L.) is one of the most important cereal crops in the world [1]. Color formation is an important part of maize kernel development, and yellow kernels are the dominant maize planted in the world [2]. Colored maize has received attention due to its role in diet-related chronic diseases [3]. Therefore, the market has different positioning demands for different colors of maize, which can be processed into different products [4]. Preferences for different maize products were important for almost all consumer (97.3%) stated diets. Maize kernels contain rich nutrients and have varying degrees of stress resistance protection for animal and human health [5].

Flavonoids, mainly anthocyanins, are the main controlling substances that determine the pigment of maize kernels [6], and mainly control the blue, purple, and red hues of kernels [7]. Studies have found that flavonoids are helpful for patients with inflammatory diseases, chronic diseases, and certain types of cancer due to their antioxidant activity [8]. At present, most of the studies on maize kernel pigmentation are genetic studies of conventional breeding. Certain studies have shown that purple kernel maize has higher contents of phenols, flavonoids, and proanthocyanins, while yellow maize has higher carotenoid content, and red kernel maize has higher contents of phenols [9, 10]. As a research tool, omics technology provides a link between gene sequences and visible phenotypes and can quantitatively determine a large number of metabolites in a short time via high throughput.

Metabolomics (LC-MS/MS) is an important technology for characterizing related bioactive substances (such as flavonoids, phenols, and carotenoids) in maize biodiversity [11]. The integration of data with other high-throughput omics technologies is critical to better understanding the underlying molecular mechanism of functional metabolism [12, 13]. In recent years, transcriptomics (RNA-Seq) and metabolomics have been applied to research and screen the metabolites and related key genes of various crops, fruit coloring, and quality formation [14–16]. Li et al. [17] investigated in the tissue-specific anthocyanin accumulation mechanism in aleurone layer and pericarp of two purple corn lines by using comparative transcriptome analysis to identify differentially expressed genes involved in anthocyanin accumulation. Maize kernels are full of nutrients, but there are few reports comparing the metabolome and transcriptome of maize grains of different colors and comparative studies on the biologically active compounds in maize grains and related pathways [9].

This study conducted a systematic analysis of different maize grain colors (white, yellow, red-purple, purple-black). We performed metabolomics to understand the composition and differences of the metabolite components in grains of different colors. Transcriptome analysis was used to explore the influence of maize grain quality- and color formation-related genes and their expression profiles, which can provide a theoretical basis for the dominant breeding of superior maize.

## Results

### Metabolite distribution in four varieties of maize kernels

**Fig. 1:**
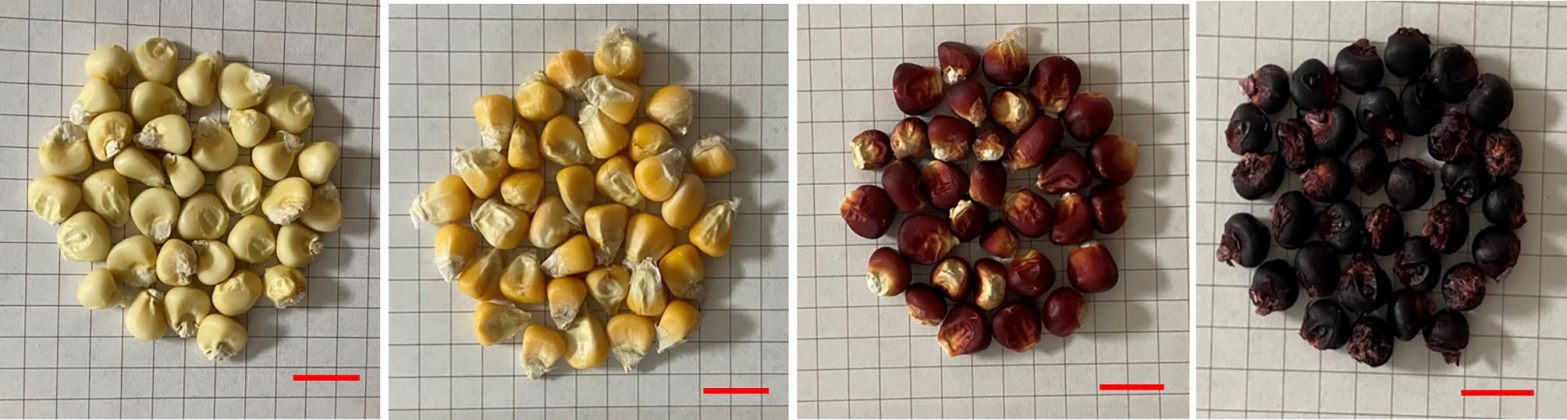
Color phenotype of four varieties of maize kernels. White (W), yellow (Y), red–purple (R) and purple–black (P) kernels from left to right in the figure. The scale in the picture represents 1 cm

**Fig. 2:**
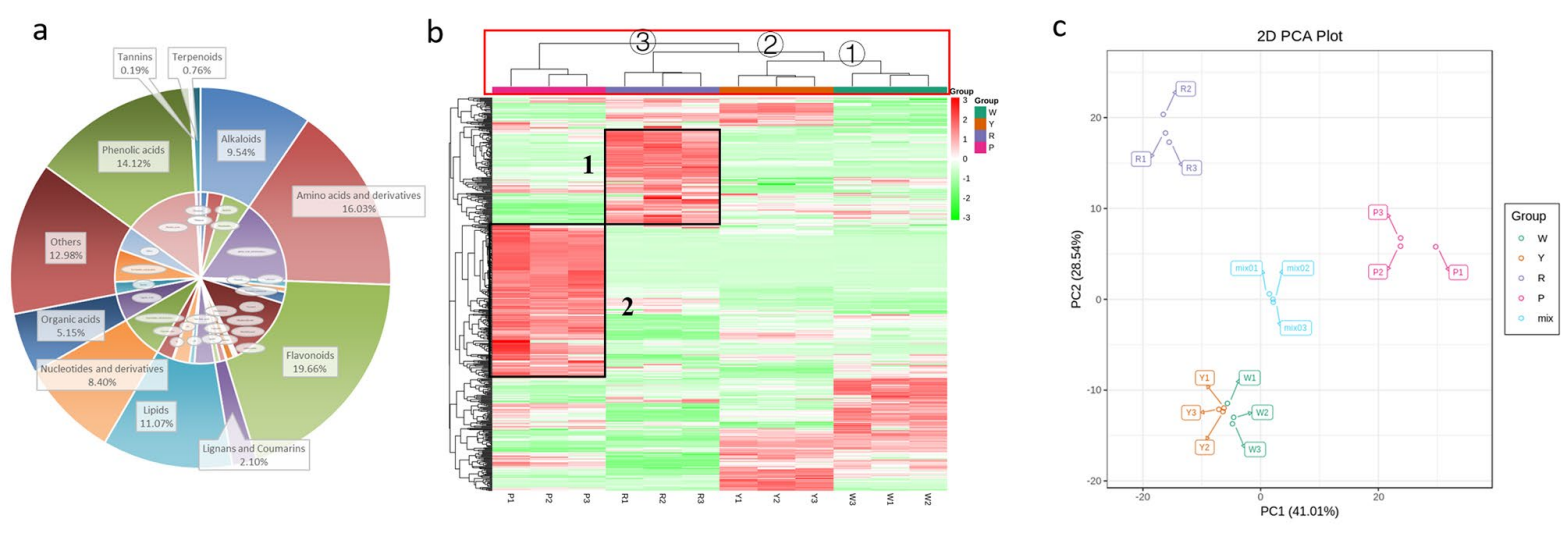
Pie chart of metabolite classification and percentages in four varieties of maize kernels (**a**). The outer circles in the pie chart indicate the detected metabolite categories and the percentage, while the inner circles show the specific subcategories of metabolites contained in each major category metabolite. Metabolite accumulation pattern of maize kernels (**b**) and principal component analysis (**c**). In the heatmap, the colors marked with green and red were down- and up-accumulated in maize kernels, respectively. W represents white kernels, Y represents yellow kernels, R represents red–purple kernels, and P represents purple–black kernels

To identify the potential mechanisms of coloration and nutritional quality between four different maize kernels with white (W), yellow (Y), red-purple (R), and purple-black (P) colors (Fig. 1). LC-MS/MS was first conducted to detect metabolites in these four varieties of maize kernels. A total of 524 metabolites were identified in this study, which mainly included flavonoids (19.66%), amino acids and derivatives (16.03%), phenolic acids (14.12%), lipids (11.07%), alkaloids (9.54%), nucleotides and derivatives (8.40%), organic acids (5.15%), and lignan and coumarin (2.10%) (Fig. 2a, Additional file 1). The results showed that these maize variety (color) kernels were rich in metabolic components.

Then, an overall analysis was conducted to understand the metabolite distribution and accumulation pattern of these components in each maize group. Figure 2b depicts hierarchical clustering analysis (HCA) for each group sample and metabolite. The results of sample clustering (red rectangular box) in the heatmap indicated that Y and W were first clustered into one branch (①) and then aggregated with R (②), while P was separated into another branch (③). This result suggested that compared with R and P, the accumulation patterns of metabolites in Y and W were closer or more similar. However, the distribution of metabolites in P and the other three groups (W, Y, and R) was quite different. The following heatmap also shows this result more intuitively (Fig. 2b). We also found that certain specific accumulated metabolites were identified in each group, especially in the R (1) and P (2) groups.

The results of the PCA were consistent with the HCA. The explained phenotypic variation rates for PC1 and PC2 were 41.01% and 28.54%, respectively. The P and R groups were significantly separated on PC1 and PC2. The Y group was close to the W group, and these two groups were located between the P and R groups on PC1 (Fig. 2c). Therefore, we combined PCA and HCA, and the results suggested that the W and Y groups had similar metabolite profiles, and their similarity to R was greater than that of the P group. The difference in metabolite distribution between the P and R groups was the largest compared with the other maize groups.

### Differentially accumulated metabolite (DAM) analysis in maize kernels

The preliminary analysis of PCA and HCA found that the metabolite accumulation and distribution in the P group was greatly different from that in the other three groups (W, R, and Y). Then, the specific differentially accumulated metabolites (DAMs) were analyzed by using the VIP > 1, -1 < log2FC < 1 criterion to filter DAMs in different comparisons, such as W vs. P, R vs. P, and Y vs. P (Fig. S1). In the comparison of R vs. P, 178 DAMs were upregulated, and 105 DAMs were downregulated. One hundred and sixty-five and 73 DAMs were upregulated and downregulated in the W vs. P comparison, respectively. In Y vs. P, 172 DAMs were upregulated, and 87 DAMs were downregulated. In W vs. R, 153 DAMs were upregulated, and 99 DAMs were downregulated. In Y vs. R, 124 DAMs were upregulated, and 121 DAMs were downregulated (Fig. S1). The number of DAMs is consistent with the previous analysis. In the above three comparison groups, the up-accumulated metabolites were significantly higher than the downregulated DAMs. The results indicated that the number of DAMs in purple maize was greater than that in other three-color maize.

### Venn analysis and shared DAMs in maize kernels

**Fig. 3:**
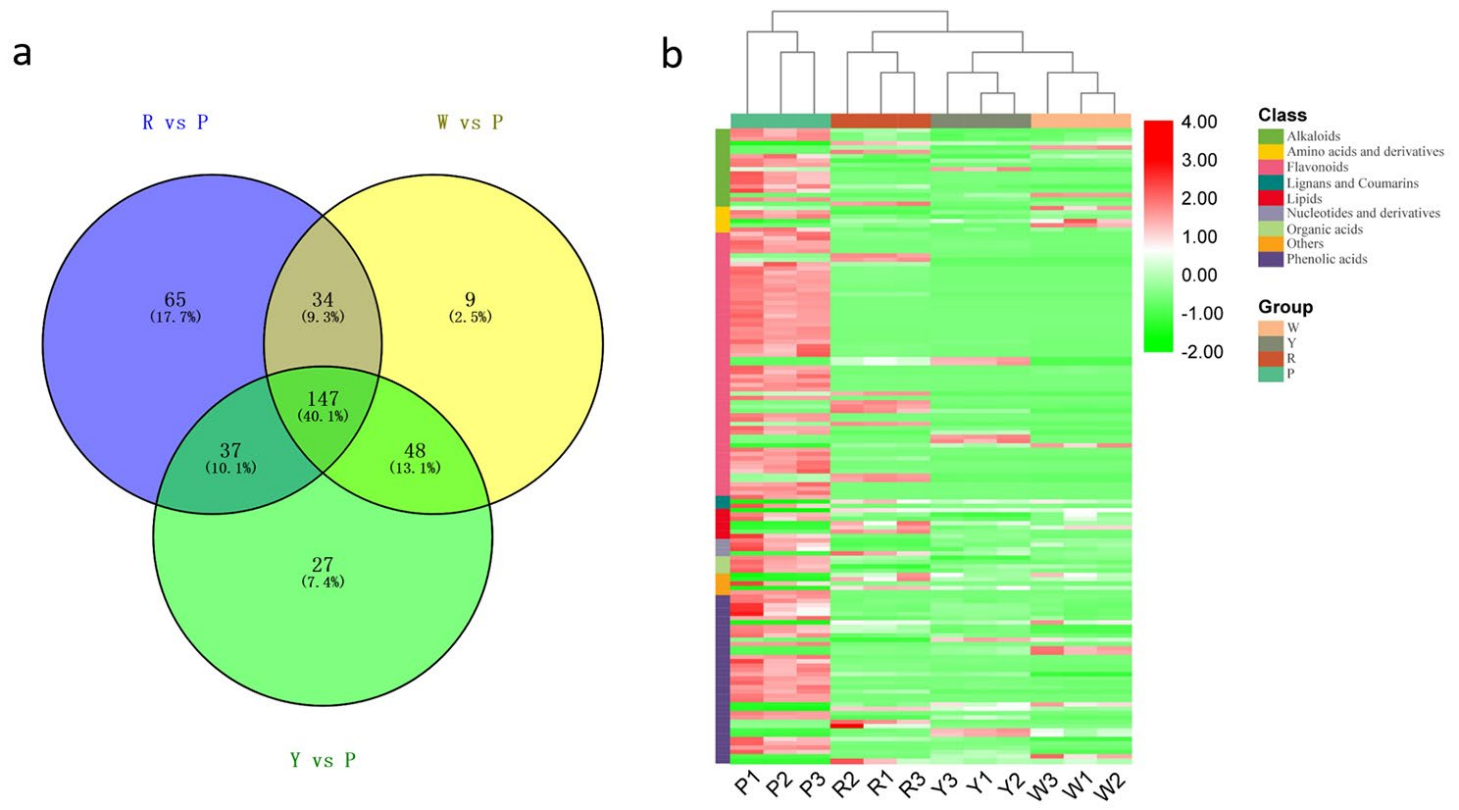
Venn analysis of identified differentially accumulated metabolites (DAMs) in maize in different comparison groups (**a**) and accumulation patterns of shared differentially accumulated flavonoids (DAFs) in four different colors of maize (**b**). W represents white kernels, Y represents yellow kernels, R represents red–purple kernels, and P represents purple–black kernels. Metabolites marked with red and green indicate up- and down-accumulated in maize kernel, respectively

To better characterize the differences between P and the other three maize groups, we used Venn analysis to screen their common ADMs (Fig. 3a). The results showed that a total of 147 DAMs were shared in each comparison maize group. In the comparison of R vs. P, Y vs. P, and W vs. P have their own unique DAMs, namely, 65, 27, and 9, respectively. Then, we carried out a detailed analysis of the content changes of these DAMs in each group of maize. Therefore, we found that the shared DAMs in the four colors of maize mainly included three categories: flavonoids, phenolic acids, and alkaloids. According to the relative content, although only a few DAMs had different accumulation patterns in each group, most DAMs were significantly accumulated in Group P.

We first conducted a detailed analysis of differentially accumulated flavonoids (DAFs) due to the largest number of flavonoids in shared DAMs (Fig. 3b, Additional file 2). A total of 61 kinds of isoflavones were identified in the present study. Among them, 46 DAFs were not detected in W, or the content was very low. In addition, 17 and 12 DAFs were not detected in the Y and R groups, respectively. However, the relative content of these DAFs in the P group was high (73%), and all of them were above 10^5^. At the same time, an anthocyanin, peonidin 3-O-glucoside, was not found in the W and Y groups, while it was highly abundant in the R (10^5^) and P groups (more than 10^7^). Since anthocyanins are important pigment-related metabolites, we speculated that the red color of maize grains in R and the purple color of kernels in P have a strong relationship with these DAFs.

We then analyzed the shared alkaloids and phenolic acids in four different colored maize kernels. The results were consistent with the accumulation pattern of flavonoids, and compared with the other three groups (W, Y, and R), these differentially accumulated alkaloids and phenolic acids were also significantly accumulated in Group P (Additional file 2). Therefore, we preliminarily speculated that the common DAMs in the W, Y, R, and P groups were mainly reflected in the secondary metabolite accumulation differences, including the accumulation of flavonoids, phenolic acids, and alkaloids.

### Accumulation patterns of specific DAMs in maize kernels

**Fig. 4:**
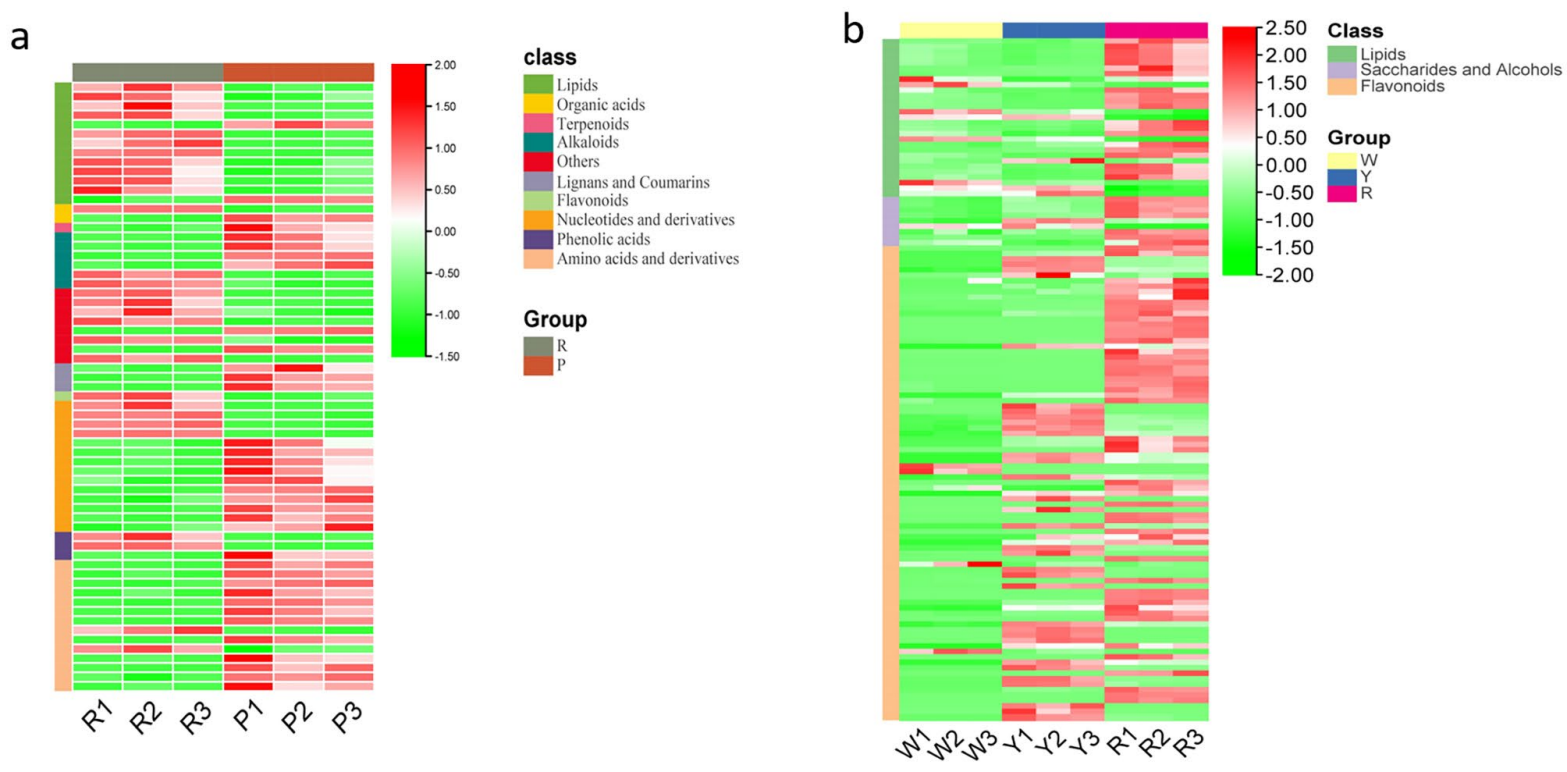
Accumulation patterns of specific metabolites (amino acids, sugar alcohols and lipids) in R vs. P (**a**); DAM (lipid, sugar alcohol, flavonoid) accumulation patterns in R vs. W and R vs. Y (**b**). W represents white kernels, Y represents yellow kernels, R represents red–purple kernels, and P represents purple–black kernels. Metabolites marked with red and green indicate up- and down-accumulated in maize kernel, respectively

According to the above HCA, PCA, and DAM screening results, we found that a huge difference existed in the R vs. P comparison. Therefore, except for the common DAMs in four color maize kernels, we were curious about the differences in specific metabolite accumulation between R and P, which contribute to the significant differences between these two kernel groups. The unique DAMs in R and P were analyzed in this study. According to the Venn analysis above (Fig. 3a), we found that a total of 65 unique DAMs in R vs. P were identified in the present study. Detailed analysis indicated that most of these DAMs (above 75%) were mainly primary metabolites, including amino acids, sugar alcohols, and lipids (Fig. 4a, additional file 3). Among them, amino acids and nucleotides notably accumulated in the P group, while sugar alcohol and lipids accumulated in the R group. Therefore, the results showed not only the accumulation difference of secondary metabolites but also the accumulation difference of primary metabolites between the R and P groups, resulting in the huge difference in DAMs between these two groups.

At the same time, we also compared the DAMs in R vs. W and R vs. Y and found the same accumulation trend in which lipids and sugar alcohol significantly accumulated in the R group (Fig. 4b). In addition to these primary DAMs, certain flavonoids also accumulated significantly in the R group (Fig. 4b, additional file 4). Finally, we analyzed the accumulation of DAMs in the W and Y groups and found that amino acids and derivatives accumulated in the W and Y groups, especially in comparisons of W vs. R and Y vs. R. Furthermore, compared with the W group, some flavonoids accumulated in the Y group as well. Therefore, we conducted significant pathway enrichment analysis (KEGG analysis) for the DAMs in W, Y groups and R, P groups, respectively. The results show that the DAMs share metabolic pathways in different comparison groups mainly including phenylpropanoid biosynthesis, phenylalanine metabolism, flavonoid biosynthesis, and flavone and flavonol biosynthesis (Fig. S3).

We analyzed the overall metabolite accumulation profile in different kernel colors of the W, Y, R, and P groups. A large number of secondary metabolites were identified in the P group, including flavonoids, phenolic acids, and alkaloids. Some secondary metabolites (flavonoids and alkaloids) also accumulated in the R and Y groups, while only a few secondary metabolites (flavonoids) accumulated in the W group, and most of these flavonoids were undetected in maize. Primary metabolites, such as amino acids and derivatives, accumulated significantly in W, while sugar alcohols and lipids accumulated significantly in red-purple kernels (R). The results suggested that the grain color from white to purple [white (W), yellow (Y), red-purple (R), and purple-black (P)] was correlated with the accumulation of flavonoids. Therefore, we speculated that these flavonoid contents were related to maize kernel color formation.

### Transcriptome analysis and KEGG enrichment analysis in each maize group

Transcriptome sequencing was performed to explore the mechanism of maize grain color differences. The RNA-Seq data are shown in Table 1. The clean bases in the 12 maize samples were all more than 6.2 G. The error rate of the sequencing did not exceed 0.02%. The Q20 and Q30 were at least 98.08% and 94.17%, respectively. The GC content of each maize group exceeded 53.99%, with at least an 86.27% read mapping rate in these samples. On the premise that the quality of RNA-Seq data was not problematic (Table 1), PCA was carried out. The transcriptome results were consistent with the metabolome PCA (Fig. S2). According to the results of metabolome analysis, we found that compared with other groups (W, Y, and R), there was a significant difference in flavonoid accumulation in Group P. Then, KEGG co-annotation was performed for differentially expressed genes (DEGs) and differentially accumulated metabolites (DAMs) identified and screened by the transcriptome and metabolome. The results showed that flavonoid biosynthesis-related pathways (flavonoid biosynthesis, flavone and flavonol biosynthesis, phenylpropanoid biosynthesis) were significantly enriched in each group of colored maize (Fig. S4). Therefore, we analyzed the associated key gene expression levels and metabolite accumulation profiles in flavonoid synthesis-related pathways.

**Table 1:** Statistics, quality and RNA-Seq assembly results of 12 RNA sequencing libraries in different color maize varieties

Sample	Raw Reads	Clean Reads	Clean Base (G)	Error Rate (%)	Q20 (%)	Q30 (%)	GC Content (%)	Reads mapped
P-1	49,714,966	48,713,390	7.31	0.02	98.26	94.64	54.56	44,390,230(91.13%)
P-2	51,957,970	50,768,754	7.62	0.02	98.47	95.17	53.99	46,294,944(91.19%)
P-3	43,694,822	42,524,096	6.38	0.02	98.25	94.59	54.8	38,643,525(90.87%)
R-1	47,480,608	46,721,244	7.01	0.02	98.14	94.31	57.07	40,522,730(86.73%)
R-2	46,743,908	45,767,870	6.87	0.02	98.34	94.93	57.21	39,486,116(86.27%)
R-3	42,128,348	41,340,490	6.2	0.02	98.25	94.68	56.69	35,925,885(86.90%)
W-1	45,094,272	44,217,510	6.63	0.02	98.23	94.52	55.6	39,729,487(89.85%)
W-2	49,294,122	48,267,112	7.24	0.02	98.28	94.64	55.24	43,486,105(90.09%)
W-3	45,642,886	44,773,192	6.72	0.02	98.08	94.17	55.2	40,259,033(89.92%)
Y-1	44,677,430	43,805,566	6.57	0.02	98.26	94.66	55.64	39,645,741(90.50%)
Y-2	47,956,890	47,250,810	7.09	0.02	98.36	94.87	55.69	42,786,601(90.55%)
Y-3	45,326,632	44,487,150	6.67	0.02	98.27	94.67	56.12	40,292,327(90.57%)

### Combined transcriptome and metabolome analysis in maize of different colors

**Fig. 5:**
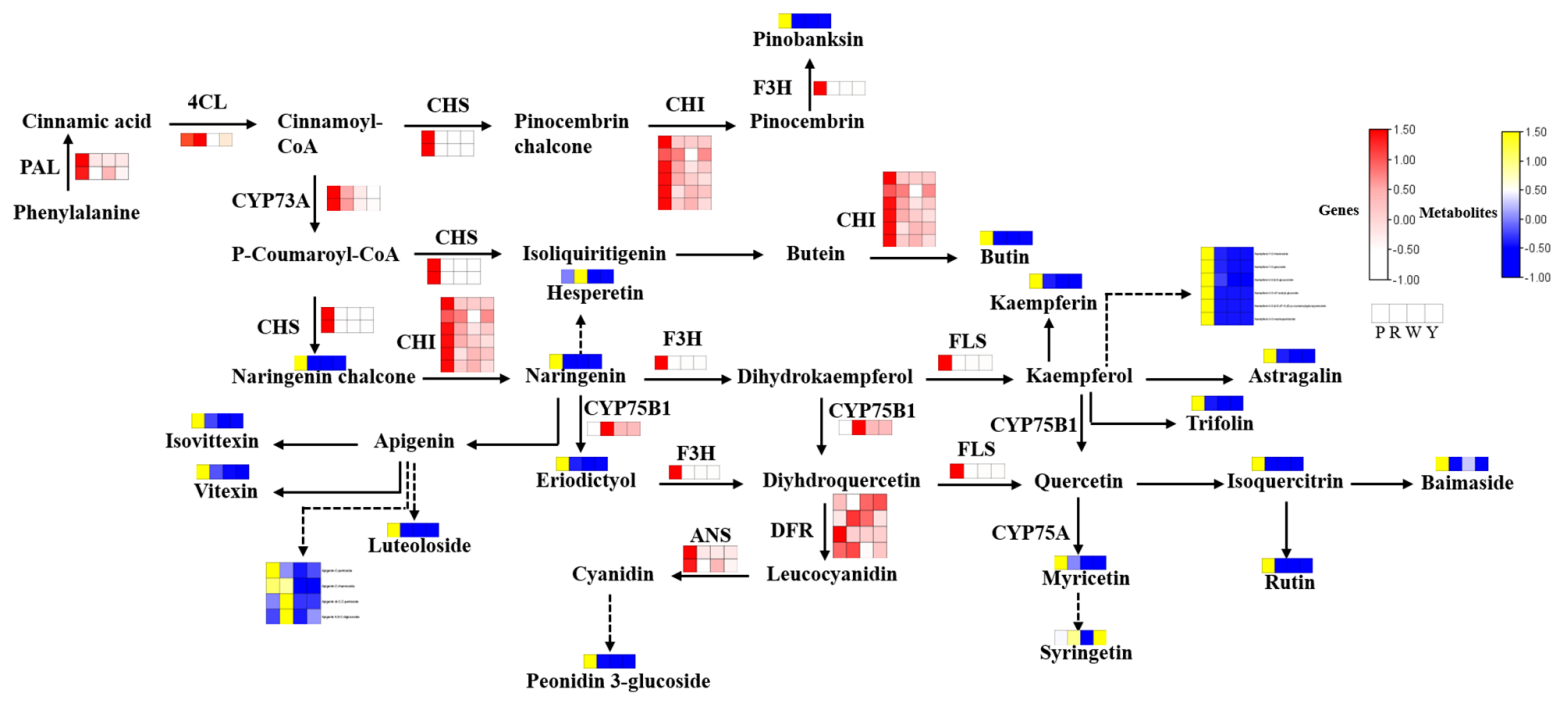
Expression levels of structural genes and metabolites involved in the flavonoid biosynthesis pathway in maize kernels. The heatmap represents the expression of corresponding genes, and white to red in the heatmap indicates the expression levels of structural genes ranging from low to high. The color of the heatmap from yellow to blue indicates the accumulation levels of metabolites ranging from low to high. Enzymes in this pathway are shown as follows: PAL, phenylalanine ammonia lyase; 4CL, 4-coumarate-CoA ligase; CHS, chalcone synthase; CHI, chalcone isomerase; CYP73A, trans-cinnamate 4-monooxygenase, F3H, flavanone 3-hydroxylase; DFR, dihydroflavonol 4-reductase; ANS, anthocyanidin synthase; FLS, flavonol synthase, CYP75A, flavonoid 3’,5’-hydroxylase, and CYP75B1, flavonoid 3’-monooxygenase

We used |log2Fold Change | ≥ 1 and FDR < 0.05 criteria to filter and identify flavonoid synthesis-related DEGs in each kernel group (R v P, W vs. P, and Y vs. P). A total of 107 DEGs were screened (additional file 5), including key synthase genes in the flavonoid synthesis pathway. According to the KEGG analysis results of DEGs and DAMs and combined with the differential flavonoid accumulation pattern (Table S2), a heatmap of the metabolic pathway flow chart was drawn in this study (Fig. 5). The results showed that flavonoids were significantly accumulated in Group P, and the expression levels of related key synthetic genes, such as *chalcone synthase* (*CHS*), *chalcone isomerase* (*CHI*), *flavanone 3-hydroxylase* (*F3H*), *phenylalanine ammonia-lyase* (*PAL*), *anthocyanidin synthase* (*ANS*), and *4-coumarate-CoA ligase* (*4CL*), were also highly expressed in Group P. In addition, the accumulation pattern of corresponding metabolites involved in regulation or synthesis by these key genes is also consistent with the gene expression profile, such as naringenin chalcone, naringenin, apigenin, luteoloside, pinobanksin, periconidin 3-glucoside, hesperetin, butin, kaempferin, kaempferol, astragalin, eriodictyol, trifolin, vitexin, isovittexin, myricetin, syringetin, rutin, isoquercitrin, and baimaside. The results of the combined transcriptome and metabolome analysis indicate that these key genes and metabolites might play an important role in the biosynthesis of flavonoids in maize kernels, which is also an important factor in determining the color formation causing the difference in the color of maize kernels.

### qRT–PCR verification

To verify the reliability of the RNA-Seq profile results, we performed qRT–PCR analysis on some key regulatory genes related to maize grain color formation (Fig. S5). These key regulated genes included *bifunctional dihydroflavonol 4-reductase*/*flavanone 4-reductase* (*DFR*), *naringenin 3-dioxygenase*, *F3H*, *CHS*, *CHI*, *chalcone-flavonone isomerase*, *leucoanthocyanidin dioxygenase/anthocyanidin synthase* (*LDOX*/*ANS*), *flavonol synthase* (*FLS*), *4CL*, and *PAL*. The results showed that qRT–PCR analysis had a significant positive correlation with the gene expression level in the RNA-Seq (R^2^ = 0.708, *P* < 0.01). Thus, the results indicate the reliability of the RNA-Seq data and the accuracy of the results of this study.

## Discussion

Maize has a high yield worldwide, and as a staple food in many countries [18], maize can have market potential and undergo further development as research on food functionality progresses. During the development of maize kernels, various metabolites are synthesized and stored [19]. Studying the molecular and biochemical mechanisms of nutritional quality and color formation in maize kernels is of great significance not only for basic research on maize development but also for maize improvement through metabolic engineering. Therefore, the dynamic transcriptome and metabolome of four varieties of maize kernels with different colors were analyzed in the present study.

Research on grain color and nutritional quality has received more attention with the development of metabonomic technology. Metabonomic analysis has been applied to a variety of grains, such as rice, wheat, and sorghum [20, 21]. However, there is a lack of comparative studies on the metabolome and transcriptome of maize kernels of different colors. In this experiment, metabolomics technology based on HPLC–MS/MS was used to study the metabolic profiles of maize kernels of different colors and to understand the differences in the principal components. A total of 524 metabolites were detected from 4 maize varieties, which mainly included flavonoids, amino acids, phenolic acids, and lipids. Among them, we found that the accumulation pattern of metabolites in group P was significantly different from that in the other three groups, while the accumulation pattern of metabolites in the Y and W groups was similar. The content and chemical diversity of flavonoids in purple maize kernels were the highest, followed by red maize, yellow maize, and kernels, indicating that the accumulation pattern and content of flavonoids might be related to the difference in maize grain color. In addition, the results of the synthesis and widespread distribution of polyphenol secondary metabolites in plants indicate that these related phenolic compounds may also be related to the coloring of grains of different colors.

As the average life expectancy of humans has increased, research on chronic diseases has become increasingly important. Flavonoids are a broad class of plant secondary metabolites that determine the color of plants or fruit skins and have also been widely used in improving animal body disease resistance and improving immune mechanisms [22, 23]. In our research, the common DAMs in maize kernels of different colors mainly included flavonoids, phenolic acids, and alkaloids. Among them, there were 61 DAFs, and as the color changed and deepened, the types increased, and their content also increased. White kernels have the least variety and the lowest content, and the difference in purple kernels is rich in flavonoids and high in content, which is consistent with previous studies [24, 25]. The results of our study showed that the type and accumulation content, and distribution ratio of flavonoids significantly affect the color of maize kernels. In addition, we also found that 46 kinds of DAFs were not detected in W, mainly including 15 flavonoids (diosmetin, 6,7,8-tetrahydroxy-5-methoxyflavone, jaceosidin, hesperetin-7-O-glucoside, 2,6-dimethyl-7-octene-2,3,6-triol, tricin 7-O-glucuronide, apigenin 6,8-C-diglucoside, lonicerin, etc.), 14 flavonols (isorhamnetin-3-O-glucoside, kaempferol-7-O-glucoside, quercetin-7-O-glucoside, isoquercitrin, etc.), and 9 dihydroflavonols (pinobanksin, hesperetin 5-O-glucoside, naringenin, butin, eriodictyol, hesperetin, and homoeriodictyol, etc.). Furthermore, 17 and 12 DAFs were not detected in Y and R, respectively, and mainly included myricetin, kaempferin, astragalin, trifolin, peonidin 3-O-glucoside, C-hexosyl-luteolin O-hexoside, eupatorine, and cynaroside. Therefore, we speculated that red–purple and purple maize grains had a strong relationship with these DAFs.

Anthocyanin, a flavonoid, is a water-soluble natural pigment widely distributed in plants [26] and is related to the main pigments found in fruits, vegetables, and flowers [27]. In this study, we found that an anthocyanin, peonidin 3-O-glucoside, was not detected in white and yellow maize kernels but had a high content in red and purple kernels. Among them, the content of peonidin 3-O-glucoside in purple maize kernels was significantly higher than that in red kernels, which may be closely related to the color change (from red–purple to purple–black) of maize grains. Previous studies have shown that this compound also has various pharmacological activities, such as antioxidative, antitumor, and cholesterol-lowering activities, and affects the immune mechanism of the animal body [28–30]. Therefore, red–purple and purple–black maize can be used as raw materials for developing functional health foods, and the different colors of maize grains can also be used as phenotypic indicators for preliminary judgment and classification.

Nutritional quality is the most important indicator of crops. In a comparison of deep-colored maize grains (R and P), 65 metabolites were found to be related to nutritional quality, such as amino acids, sugar alcohols, lipids, and lignans and coumarins, which were important factors in grain quality [31]. There were almost no differential metabolites associated with color formation because these DAMs were all present in both R and P maize kernels but with different relative contents to decide color changes. Therefore, we speculate that the difference in color between R and P is determined by the content of flavonoids/anthocyanins. Nutritional quality analysis of these two maize varieties showed that P contained more amino acids and nucleotides, while R contained more sugar alcohols and lipids. Lignans and coumarins belong to the class of phenylpropanoids that have anticoagulant, hemostasis, anticancer, and photosensitivity effects on animals [32–34], which have growth regulation and disease resistance effects on plants. In addition, coumarin has a sweet smell that may contribute to the flavor modification of maize kernels. The results suggested that the two maize varieties had different nutritional values and taste sensations.

## Conclusion

Transcriptomics data can be combined with metabolic networks that clarify the reasons for different maize kernel colors and provide metabolic network information for subsequent customized cultivation of functionally enriched maize. The results of metabolomic and transcriptomic analysis showed that DEGs and their metabolites were significantly enriched in flavonoid synthesis pathways. Compared with the light-colored maize kernels, the levels of these compounds in the dark-colored maize kernels were significantly higher. Flavonoids and anthocyanins are important pigment-related metabolites. Therefore, the different accumulation of flavonoids made different color of the maize grains in group R (red), group Y (yellow), and group P (purple). At the same time, amino acids, nucleotides, sugar alcohols and lipids accumulate specifically in P and R to form nutritional qualities. The expression levels of key flavonoid/anthocyanin biosynthesis-related genes were also consistent with the metabolite accumulation pattern in these different colored maize kernels, and the reliability was verified by qRT–PCR analysis. Our results showed that there are considerable molecular (transcriptional and metabolic) changes in colored maize grains, thus revealing the potential regulation of nutritional quality and color formation in maize kernels.

## Materials and methods

### Plant materials

White waxy maize (W), yellow waxy maize (Y), red-purple waxy maize (R), and purple-black waxy maize (P) were selected in this experiment (Fig. 1). Among them, W was derived from the inbred line of Xuannuo 255. Y is derived from the inbred line of Xiandanuo 001. R was derived from the inbred lines of hybrid Jingfeng 5 and Peruvian black maize after continuous self-breeding. P was derived from the inbred lines of hybrid Jingfeng 5 and hybrid Jingnuo 308. The four maize varieties are cultivated in the same soil environment and with the same planting crop technique at the Guangxi Academy of Agricultural Sciences Experiment Station in Guangxi, China. The maize plants are arranged according to a random block design. Each variety was grown in a single row 3 m × 0.6 m in length and width, with 10 individual maize plants per row. Isolation zones are designed between each variety to ensure that different maize varieties were self-pollinated when more than 80% silk appeared. The waxy ripening (close to physiological maturity) of fresh maize/corn kernels were sampled within 25–28 days after pollination. The sampling time was November 26, 2019, and the location was Mingyang Base of Guangxi Zhuang Autonomous Region, Nanning Jiangnan District, Academy of Agricultural Sciences (CAAS) (N: 22°36’ 28.69”, E: 108°14’ 4.16”). In this study, three replicates were made for each group of maize samples. In order to ensure the authenticity and accuracy of the samples and reduce systematic error in the experiment, a replicate consisted of more than 6 maize plants with the same growth pattern from each maize variety. All of the maize kernels (W, Y, R, and P) were placed in a centrifuge tube and quickly frozen in liquid nitrogen to brought back to the laboratory. Then transferred these samples to a -80℃ refrigerator for subsequent LC-MS/MS and RNA-Seq detection.

### Determination of metabolite content

Twelve samples from different colored maize kernel cultivars (W, Y, R, and P maize kernels) were washed three times with ddH_2_O and freeze-dried for the subsequent test. Three biological replicates were used for all samples. A single biological replicate of each maize variety was collected from more than 10 maize plants and fully mixed. Each colored maize kernel had an exact 100 mg of powder using a grinder to the ground for 1.5 min at 30 Hz. The collected extraction samples were obtained to acquire the content of metabolites in Wuhan Metware Biotechnology Co., Ltd. (www.metware.cn) [35]. The extraction simplification step and conditions were as follows: 1 ml 70% v/v precooled (4℃) methanol was added to a 2.0 ml microcentrifuge tube with a 100 mg sample of powder stored at 4 °C overnight for extraction, centrifuged at 10,000 g for 10 min and then the supernatants were immediately acquired. CNWBOND Carbon-GCB SPE Cartridge (250 mg, 3 ml) was used; ANPEL (Shanghai, China) was subsequently absorbed. Then, the harvested extracts were further analyzed in the following section. Sample extracts were analyzed using an LC–ESI–MS/MS system (HPLC, Shimpack UFLC SHIMADZU CBM30A system; MS, Applied Biosystems 6500 Q TRAP; MS, API 6500 Q TRAP) [36].

### Analysis of total metabolite content

All metabolite identification was annotated by the MetWare database platform (MWDB), which is used to detect more than 5000 substances, covering more than 16 categories of metabolites. The relative content of substances was accurately detected by triple and four-stage MRM mode, including an ultra-performance liquid chromatography system (UPLC), a tandem mass spectrometry system (MS/MS), and a partial least squares discriminant analysis of the samples [36, 37]. For quantitative accuracy detection of the relative content of substances acquired on a triple quadrupole-linear ion trap mass spectrometer (Q TRAP), LIT and triple quadrupole (QQQ) scans were used.

### RNA isolation and transcriptome analysis

The RNA of each color maize kernel was isolated from 4 different colored maize kernels using the mirVana miRNA isolation kit, using 1% agarose gels to detect the RNA’s degradation and contamination, using an RNA Nano 6000 Assay Kit of the Bioanalyzer 2100 system (Agilent Technologies, CA, USA) to verify the RNA’s quality. Total RNA was extracted from the maize kernels and reverse transcribed to cDNA using the RNAprep Pure Plant Kit (Tiangen Biotech, Beijing, China). The cDNA library was sequenced on an Illumina sequencing platform (HiSeqTM 2500), and the original data were subsequently processed to acquire clean reads by using Trimmomatic [38]. Finally, more than 41,340,490 clean reads were assembled into contigs, and these contigs were assembled into transcripts using Trinity in the paired-end method [39]. The integrative analysis between metabolites and genes in the flavonoid biosynthesis pathway used the Spearman correlation test to select the coefficient data that satisfied the standard with a P-value < 0.05 and R > 0.9. The database resources TBTools and Cytoscape were used to process the data. Classified protein and annotated protein functions and pathways of the genes were determined by the UniProt-GOA database (http://www.ebi.ac.uk/GOA/), the Gene Ontology (GO) (http://www.geneontology.org/), the NCBI (https://www.ncbi.nlm.nih.gov/), GO, and KEGG databases [40]. Using DESeq2 software for each sample’s differentially expressed gene (DEG) analysis, the false discovery rate (FDR) was set to meet conditions < 0.05 and |log2 FC| of ≥ 1.

### Real-time polymerase chain reaction (qRT–PCR) analysis

An SYBR Green PCR kit (Qiagen, Dusseldorf, Germany) containing 5 µL 2xSYBR Green mix, 1 µL cDNA, 0.5 µL forward primer, 0.5 µL reverse primer, and 3 µL ribonuclease (RNase)-free water was used to test the gene expression level. The qRT-PCR system conditions were as follows: 95 °C for 2 min and 38 cycles of 95 °C for 15 s, 58 °C for 15 s, and 72 °C for 40 s [16]. Gene-specific primers were used for analysis. The designed primers are shown in Table S1. The actin gene was used as the normalized reference gene for all tested transcripts, and the expression of genes was calculated. The differential expression of genes was tested using the formula F = 2^−ΔΔCt^. All samples were repeated three times. qRT–PCR data were analyzed by R software 3.1.3 (http://cran.r-project.org/), and the FPKM (fragments per kilobase of transcript per million) value was normalized using log2 (fold change) measurements.

### Statistical analysis

All the experiments in this study had three biological replicates. Data analysis was performed using the tools included GraphPad Prism 5 and SPSS v20.0 (SPSS Inc., Chicago, IL, USA). The partial least squares-discriminant analysis (PLS-DA) model and Analyst 1.6.1 software were used to analyze the metabolite data and check the ed variable importance in projection (VIP) value. Identification of VIP scores ≥ 1 and |log2 (fold change) | ≥ 1 as differentially metabolite for subsequent data analysis. The threshold P < 0.05 was considered to be statistically significant. VIP > 1, -1 < log2FC < 1 criterion was used to filter the specific differentially accumulated metabolites (DAMs) between different colored maize variety and − 1 < log2FC < 1, FDR < 0.05 criteria to filter and identified differentially expressed genes(DEGs)in different comparisons group.

## Electronic supplementary material

Below is the link to the electronic supplementary material.

Supplementary Material 1

Supplementary Material 2

Supplementary Material 3

Supplementary Material 4

Supplementary Material 5

Supplementary Material 6

Supplementary Material 7

## Data Availability

The datasets analyzed during the current study are available in the [NCBI BioProject repository, PRJNA902041].
