# Supplementary material for: Metabolomics and transcriptomics strategies to reveal the mechanism of diversity of maize kernel color and quality
Source: BMC Genomics. 2023 Apr 12;24:194. doi: 10.1186/s12864-023-09272-x (PMC10091680; doi:10.1186/s12864-023-09272-x)
Supplement: Supplementary file 6 — Supplementary Material 6 [file 12864_2023_9272_MOESM6_ESM.docx]

Table S1. List of primers used by qRT-PCR.

| **Gene-ID** | **Chr** | **F (5'-3')** | **R (5'-3')** | **Products (bp)** | **Tm (℃)** |
| --- | --- | --- | --- | --- | --- |
| Zm00001e020384 | chr3 | CAGGGATAGGTACCCCGAGT | TCCTCCAGCGTCTTGTACCT | 132 | 56 |
| Zm00001e006649 | chr2 | TTCTCCACTACCGGTCCATC | TGCTGCTTCTGCTGCTGTAT | 161 | 55 |
| Zm00001e024218 | chr4 | TGCTTGGTTCCTTCAATTCC | GAGAGACCGATCGAGCTAGG | 102 | 54 |
| Zm00001e020880 | chr3 | ACCGCAAGCTCAAGTGTCTT | TTTTCACCTGGTGTGTTGGA | 110 | 55 |
| Zm00001e023411 | chr4 | GAAGCTTGTGGAGGGTTTCA | GAGCATCAGGTGTTTCAGCA | 96 | 57 |
| Zm00001e038238 | chr9 | ATCCCGATGAGCGTGTAGAG | AAAGCTCCTCACGAAGTCCA | 95 | 58 |
| Zm00001e012906 | chr5 | TCTCTGCTTGCCAGGAATCT | TCTTTGCAGTTGCATTCAGG | 139 | 56 |
| Zm00001e006178 | chr1 | AGAAGTTCACGAGGGTGACG | AGTGCGTGAAGAGGATGGAG | 195 | 55 |
| Zm00001e011164 | chr2 | CCGACTGGAACTCCATCTTC | ATGTTGCCGTACTCGGAGAG | 133 | 54 |
| Zm00001e002816 | chr1 | TGGAGCTTGGCGAAGACTAT | TGGACGGAACAGTGTACCAA | 213 | 57 |
| Zm00001e037729 | chr9 | GGCCACAAATGTGAAGACCT | GTAGTTGCCCCACGTGTTCT | 90 | 55 |
| Zm00001e013798 | chr5 | GTGTTCACTGAGGCCCGTAT | CCCTTCTGTTGCCAGTTCAT | 113 | 57 |
| Zm00001e007572 | chr2 | GCTCTTCTCCACTCCACGTC | GGTGAGCTGGGAAGTTCTGA | 91 | 55 |
| Actin-1 | - | GCCTACGTTGCCCTTGATTA | ACCTGACCATCAGGCATCTC | 92 | 58 |

Table S2. Detailed information of metabolites significantly enriched in flavonoid biosynthesis pathways in four color maize varieties.

| **Index** | **Q1 (Da)** | **Q3 (Da)** | **Molecular Weight (Da)** | **Formula** | **Ionization model** | **Compounds** | **CAS** |
| --- | --- | --- | --- | --- | --- | --- | --- |
| pme2960 | 273.08 | 153 | 272.068 | C15H12O5 | [M+H]+ | Naringenin chalcone | 73692-50-9 |
| pme0376 | 271.06 | 151 | 272.068 | C15H12O5 | [M-H]- | Naringenin | 480-41-1 |
| pme3475 | 273.08 | 153 | 272.068 | C15H12O5 | [M+H]+ | Butin | 492-14-8 |
| mws0914 | 271.06 | 151 | 272.068 | C15H12O5 | [M-H]- | Pinobanksin | 548-82-3 |
| mws0064 | 287.06 | 135 | 288.063 | C15H12O6 | [M-H]- | Eriodictyol | 552-58-9 |
| mws0463 | 301.07 | 164 | 302.079 | C16H14O6 | [M-H]- | Hesperetin | 520-33-2 |
| mws0032 | 319.04 | 153 | 318.038 | C15H10O8 | [M+H]+ | Myricetin | 529-44-2 |
| mws1002 | 345.06 | 330 | 346.069 | C17H14O8 | [M-H]- | Syringetin | 4423-37-4 |
| mws1434 | 431.1 | 311 | 432.106 | C21H20O10 | [M-H]- | Apigenin-6-C-glucoside (Isovitexin) | 29702-25-8 |
| mws0919 | 431.1 | 285 | 432.106 | C21H20O10 | [M-H]- | Kaempferol-3-O-rhamnoside (Afzelin) (Kaempferin) | 482-39-3 |
| mws0048 | 431.1 | 311 | 432.106 | C21H20O10 | [M-H]- | Apigenin-8-C-Glucoside (Vitexin) | 3681-93-4 |
| mws0913 | 447.09 | 285 | 448.101 | C21H20O11 | [M-H]- | Kaempferol-3-O-galactoside (Trifolin) | 23627-87-4 |
| pme2459 | 449.11 | 287.2 | 448.101 | C21H20O11 | [M+H]+ | Luteolin-7-O-glucoside (Cynaroside) | 5373-11-5 |
| mws2209 | 449.11 | 287.06 | 448.101 | C21H20O11 | [M+H]+ | Kaempferol-3-O-glucoside (Astragalin) | 480-10-4 |
| mws0091 | 463.09 | 300.03 | 464.095 | C21H20O12 | [M-H]- | Quercetin-3-O-β-D-glucoside (Isoquercitrin) | 482-35-9 |
| mws0059 | 609.15 | 301 | 610.153 | C27H30O16 | [M-H]- | Quercetin-3-O-rutinoside (Rutin) | 153-18-4 |
| Lmyn001226 | 625.14 | 301.06 | 626.149 | C27H30O17 | [M-H]- | Quercetin-3-O-β-D-sophoroside | 18609-17-1 |
| pmf0203 | 463.12 | 301.07 | 463.124 | C22H23O11+ | [M]+ | Peonidin 3-O-glucoside | 68795-37-9 |
| HJAP014 | 403.09 | 367.08 | 402.095 | C20H18O9 | [M+H]+ | Apigenin-C-pentoside | - |
| Hmgp003664 | 417.12 | 381.1 | 416.11 | C21H20O9 | [M+H]+ | Apigenin-C-rhamnoside | - |
| pme0321 | 431.1 | 285 | 432.106 | C21H20O10 | [M-H]- | Kaempferol-7-O-rhamnoside | 20196-89-8 |
| mws0089 | 447.09 | 285.04 | 448.101 | C21H20O11 | [M-H]- | Kaempferol-7-O-glucosdie | 16290-07-6 |
| Lmzn001894 | 461.07 | 285.04 | 462.08 | C21H18O12 | [M-H]- | Kaempferol-3-O-β-D-glucuronide | - |
| Zmhn003565 | 489.1 | 285.04 | 490.111 | C23H22O12 | [M-H]- | Kaempferol-3-O-(6''-acetyl)-glucoside | - |
| pmb0654 | 535.14 | 481.1 | 534.137 | C25H26O13 | [M+H]+ | Apigenin di-C, C-pentoside | - |
| Lmmp003217 | 595.14 | 287.06 | 594.137 | C30H26O13 | [M+H]+ | Kaempferol-3-O-β-D-(6''-O-(E)-p-coumaroyl) glucopyranoside | - |
| Lmbp003230 | 595.17 | 287.05 | 594.158 | C27H30O15 | [M+H]+ | Kaempferol-3-O-neohesperidoside | 32602-81-6 |
| mws1073 | 595.17 | 457.2 | 594.158 | C27H30O15 | [M+H]+ | Apigenin 6,8-C-diglucoside | 23666-13-9 |
