# Supplementary material for: Metabolomics and transcriptomics strategies to reveal the mechanism of diversity of maize kernel color and quality
Source: BMC Genomics. 2023 Apr 12;24:194. doi: 10.1186/s12864-023-09272-x (PMC10091680; doi:10.1186/s12864-023-09272-x)
Supplement: Supplementary file 7 — Supplementary Material 7 [file 12864_2023_9272_MOESM7_ESM.pdf]

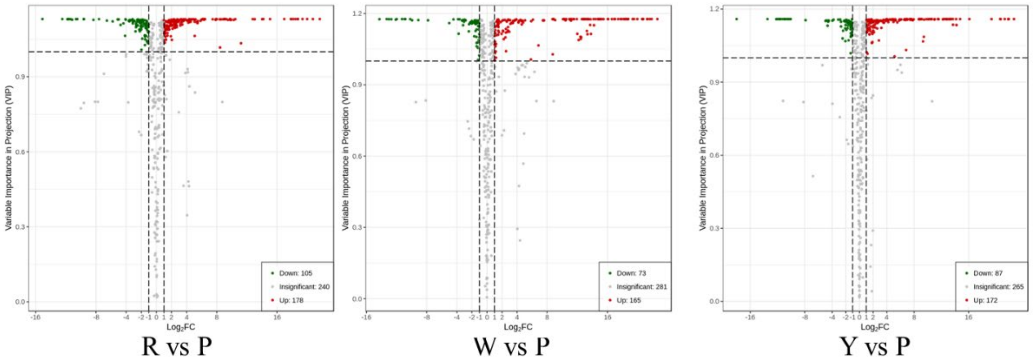

Figure S1. Volcano plots of differentially accumulated metabolites from different comparison maize groups. The green dots represent down-accumulated metabolites, and the red dots represent up-accumulated metabolites between different caparisons. W represent white kernels, Y was yellow kernels, R was red-purple kernels, P was purple-black kernels.

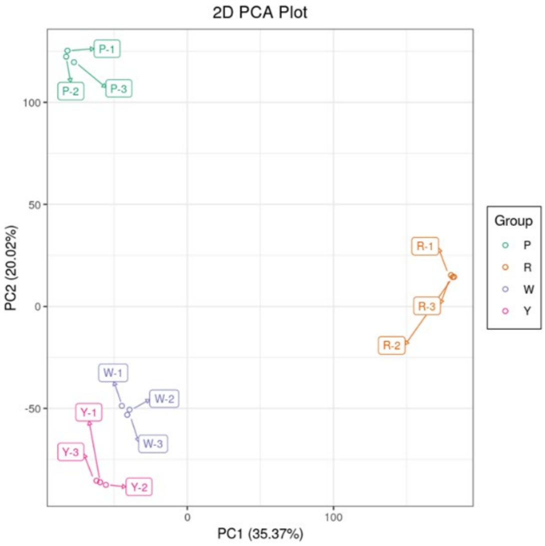

Figure S2. Principal component analysis (PCA) of RNA-Seq data in four different colors maize varieties. W represent white kernels, Y was yellow kernels, R was red-purple kernels, P was purple-black kernels.

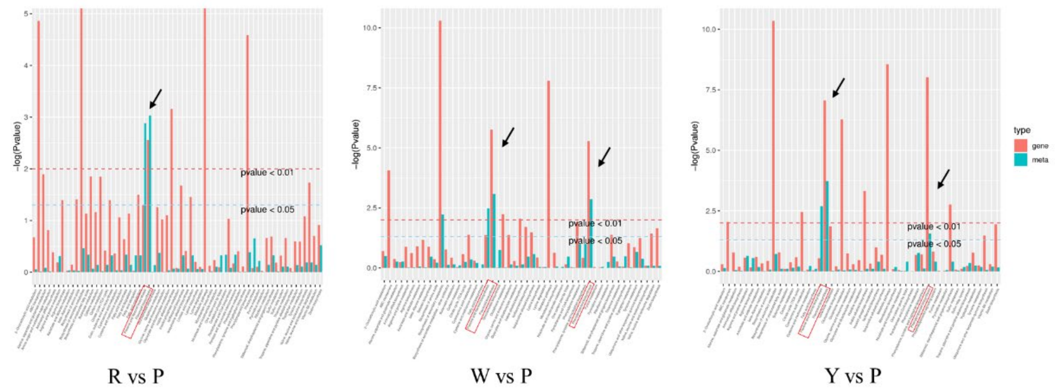

Figure S3. Combined analysis of metabolome and transcriptome data of maize with four different colors. The pathways correlated with differentially expressed genes (DEGs) and DAMs in different maize groups (Flavonoid biosynthesis, flavonol and flavonol biosynthesis, phenylpropanoid biosynthesis). W represent white kernels, Y was yellow kernels, R was red-purple kernels, P was purple-black kernels.

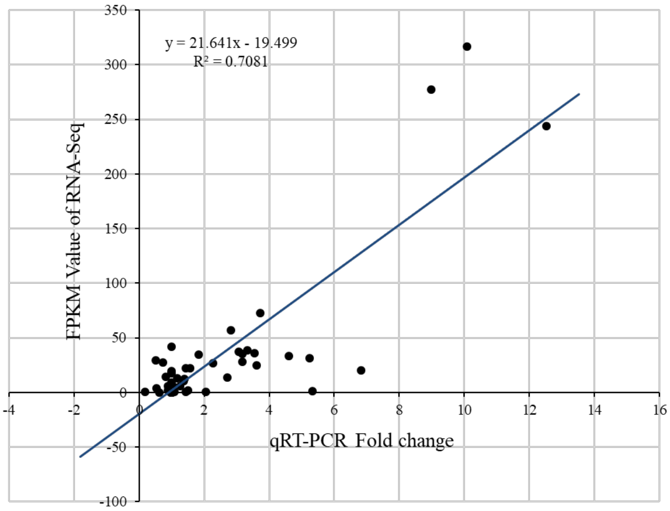

Figure S4. qRT-PCR validation and transcriptome data analysis.
